# Current availability and status of paediatric cardiac transplantation and mechanical circulatory support in twenty-eight European countries

**DOI:** 10.1007/s00431-026-06927-1

**Published:** 2026-04-15

**Authors:** Oscar van der Have, Esme Dunne, Karin Tran-Lundmark, Julie Wacker, Ina Michel-Behnke, Karel Koubsky, Annemarie Krauss, Johan Van Cleemput, Sigurdur Sverrir Stephensen, Michiel Dalinghaus, Dorotea Šijak, Mirko Topalovic, Inguna Lubaua, Zulal Ulger, Vladimiro Vida, Klaus Juul, Christian Balmer, Senka Mesihovic-Dinarevic, José Fragata, Bohdan Maruszewski, Taisto Sarkola, László Ablonczy, Iolanda Muntean, Martin Zahorec, Milind Chaudhari, Thomas Möller, Damien Bonnet, Jacob Simmonds, Zdenka Reinhardt, Colin J. McMahon

**Affiliations:** 1 Department of Experimental Medical Science and Wallenberg Center for Molecular Medicine, Lund University, Lund, Sweden; 2 The Paediatric Heart Center, Skåne University Hospital, Lund, Sweden; 3 Department of Paediatric Cardiology, Children’s Health Ireland, Crumlin, Ireland; 4 Paediatric Cardiology Unit, Department of Paediatrics, Gynaecology, and Obstetrics, Geneva University Hospitals, Geneva, Switzerland; 5 Department of Paediatrics and Adolescent Medicine, Medical University of Vienna, Vienna, Austria; 6 Children’s Heart Centre, Second Faculty of Medicine, Charles University, Motol and Homolka University Hospital, Prague, Czech Republic; 7 Department of Congenital Heart Disease, Paediatric Cardiology, Deutsches Herzzentrum der Charité, Berlin, Germany; 8 Department of Cardiology, University Hospitals Leuven, Leuven, Belgium; 9 Children’s Hospital, University Hospital of Iceland, Hringbraut, Reykjavik, Iceland; 10 Department of Paediatric Cardiology, Sophia Children’s Hospital, Erasmus University Medical Center, Rotterdam, The Netherlands; 11 University Hospital Centre, Zagreb, Croatia; 12 Cardiology Department, Paediatric Clinic, University Medical Centre Ljubljana, Ljubljana, Slovenia; 13 Department of Paediatric Cardiology, Riga Stradins University, Riga, Latvia; 14 Department of Paediatric Cardiology, Faculty of Medicine, Ege University, İzmir, Turkey; 15 Paediatric Cardiac Surgery, Department of Cardiac, Thoracic, Vascular Sciences and Public Health, University of Padua, Padua, Italy; 16 From the Department of Paediatrics and Adolescent Medicine, Copenhagen University Hospital, Rigshospitalet, Copenhagen, Denmark; 17 Division of Paediatric Cardiology, Paediatric Heart Center, Department of Surgery, University Children’s Hospital Zurich, Zurich, 8032 Switzerland; 18 Committee for Cardiovascular Pathology, Academy of Sciences and Arts Bosnia and Herzegovina, Saravejo, Bosnia and Herzegovina; 19 Department of Cardiothoracic Surgery & Transplantation, Hospital de Santa Marta, Centro Hospitalar Universitário Lisboa Central, Lisbon, Portugal; 20 Department of Cardiothoracic Surgery, Children’s Memorial Health Institute, Warsaw, Poland; 21 Children’s Hospital, Paediatric Research Center, University of Helsinki, and Helsinki University Hospital, Helsinki, Finland; 22 Gottsegen National Cardiovascular Center, Budapest, Hungary; 23 Department of Paediatric Cardiology, Emergency Institute for Cardiovascular Diseases and Heart Transplant, Pharmacy, Sciences and Technology of Targu Mures, George Emil Palade University of Medicine, Targu Mures, Romania; 24 Department of Paediatric Cardiology, Comenius University, Bratislava, Slovakia; 25 Department of Paediatric Cardiology, Birmingham Women’s and Children’s NHS Foundation Trust, Birmingham, UK; 26 Department of Paediatric Cardiology, Oslo University Hospital, Oslo, Norway; 27 Unité Médico-Chirurgicale de Cardiologie Congénitale et Pédiatrique Centre de Référence Malformations, Cardiaques Congénitales Complexes - M3C Hôpital Necker Enfants Malades, APHP Université Paris Descartes, Sorbonne Paris Cité, Paris, France; 28 Department of Paediatric Cardiology, Great Ormond Street Hospital for Children, London, UK; 29 Freeman Hospital, Newcastle upon Tyne, UK; 30 School of Medicine, University College Dublin, Belfield, Dublin 4 Ireland

**Keywords:** Paediatric heart transplantation, Ventricular assist device, Association for European Paediatric and Congenital Cardiology, Practice heterogeneity

## Abstract

We sought to investigate the inter- and intra-country variation in paediatric heart transplantation (HTx) and mechanical circulatory support (MCS) availability and practice in Europe. Data was obtained through a survey circulated to paediatric transplant cardiologists identified through the Association of European Paediatric and Congenital Cardiology between November 2022 and March 2023. Twenty-eight respondents from twenty-eight European countries completed the survey. Twenty-four (86%) had paediatric and 25 (89%) had adult congenital HTx services. National paediatric HTx centre density ranged from 0.00 to 5.35 per 10 million inhabitants. Eighteen (64%) countries followed either Eurotransplant or Scandiatransplant protocols for organ allocation and 59% of the surveyed countries had low HTx volumes (< 4 paediatric HTx annually), based on the self-reported numbers. A ventricular assist device (VAD) programme was operational in 22 (79%) countries. In only 9 countries (38%), there were dedicated resources for HTx as part of the hospital budget; however, a vast majority (77%) reported that families suffered no cost when a VAD was utilised. Twenty-one countries (78%) reported limited intensive care unit beds and 14 (54%) reported that HTx impacted on congenital heart surgery cases. Only 12 of 27 countries (44%) had a standardised national protocol for post-HTx care. There was no correlation between paediatric HTx centre density, population or GDP per capita.

*Conclusion*: There is a high degree of variation both within and between the surveyed European countries with regard to paediatric HTx centre density, VAD availability, listing protocols, and post-HTx management. Multiple factors contribute to this heterogeneity, which makes standardisation of HTx listing and VAD criteria challenging. Increased cross-national collaborative efforts between European countries may strengthen both HTx and MCS availability and outcomes, especially in regions with several smaller neighbouring countries.

**Table:** 

**What is Known:** • *The care of paediatric heart transplant (HTx) and mechanical support (MCS) patients varies across Europe.*	
**What is New:** • *This paper elucidates the differences in transplant service organisation and the care of transplant and MCS patients in twenty-eight European countries.* • *These findings could potentially encourage broader open dialogue and facilitate collaboration across European paediatric HTx centres with development of standardised listing criteria, improved collective European registry data and creation of standards for screening post-HTx patients.*

**Supplementary Information:**

The online version contains supplementary material available at 10.1007/s00431-026-06927-1.

## Introduction

Paediatric heart failure is a disease entity distinct from that of the adult disease, encompassing a heterogenous group of patients and underlying aetiologies [1]. It is associated with significant mortality; a US-based study from 2022 revealed a 4.3% mortality associated with paediatric heart failure hospitalisations [2]. In comparison with adult heart failure patients, hospitalisations for children have shown to incur roughly three times the cost [2, 3]. Heart transplantation (HTx) in children is a widely accepted palliative treatment in the setting of end-stage heart failure [4], albeit donor shortage continues to be a cause of significant waiting list mortality in infants and younger children.

Collation of multicentre data provides valuable insights over retrospective single centre data in this patient population. Collective data comes from a few primary sources; the registry of the International Society for Heart and Lung Transplantation (ISHLT), the Paediatric Heart Transplant Society (PHTS) and the National Health Service Blood and Transplant in addition to smaller pooled studies. Up until June 2017, 15,726 paediatric heart transplants had been reported to the ISHLT registry, of which 25% had been performed in Europe [5]. Differences in the paediatric HTx populations of Europe and North America are well-characterised; Europe has a lower percentage of congenital heart disease patients referred for HTx, oftentimes an older median age at HTx and lists fewer infants under the age of 1 year [6–11]. Extrapolation of data from the aforementioned registries might therefore provide guidelines that are sub-optimal for European paediatric HTx conditions and practices.

Given the heterogeneity between paediatric HTx recipient populations in North America and Europe, the increased complexity and burden of disease prior to HTx and the increased use of mechanical circulatory support (MCS) [10, 12, 13], surveying practitioners across Europe could help us understand how countries have adapted to the new era of paediatric HTx. This might also identify gaps in availability and highlight challenges in current clinical practice. Additionally, there is a dearth of information related to changes in paediatric HTx since the COVID-19 pandemic [14]. The aim of this study was to survey practice variation and availability, correlate such variation with population and gross domestic product (GDP), and to define real-life challenges faced by practitioners in the more recent era.

## Materials and methods

### Study and survey design

This was a multinational, multicentre study surveying lead paediatric transplant cardiologists in Europe on availability, practice patterns, and pandemic adaptations in the field of paediatric MCS and HTx. A survey (Supplemental Information 1) was generated using SurveyMonkey (SurveyMonkey Inc, San Mateo, California, USA) and underwent detailed review by the committee of the Association for European Paediatric and Congenital Cardiology (AEPC) pulmonary hypertension/heart failure/transplant group. The survey consisted of 49 questions with both categorical answers and free-text responses. Informed consent was obtained from all participants, and the survey was not anonymous. All responses were obtained between the November 26th 2022 and March 21st 2023.

Respondent demographics were collected, including centre, geographic location, country of origin, and presence or absence of paediatric HTx services. Further information was then collected on the type of HTx services available, approximate volume of transplants performed per year and types of ventricular assist device (VAD) services available. Transplant centre volumes were defined as low (average of < 4 transplants annually), intermediate (average of 4–11.99 transplants annually), and high (average of ≥ 12 transplants annually) as previously described [14]. The remaining questions focused on four main areas of practice: transplant listing, VAD, post-transplant care, and allocation of resources in paediatric HTx practice.

### Survey distribution

Requests for survey participation were sent via e-mail to lead paediatric cardiologists with special interest in heart failure, VAD, and HTx management, identified via the AEPC national delegate in each AEPC member country (*n* = 33) or through members of the pulmonary hypertension, heart failure, and transplantation working group within the AEPC. If no response was obtained, a total of two reminders were sent via email to encourage participation.

### Ethical approval

Ethical approval for this study of paediatric HTx practitioners was waived through the local ethics committee at Children’s Health Ireland in Crumlin. All participants gave their informed consent before partaking in the survey, and this study was conducted in accordance with the Declaration of Helsinki.

### Statistical analysis

Only completed surveys were included in the analysis. Descriptive statistical analyses were used to present the data. Data were summarised as means (standard deviation) or medians (interquartile ranges) as appropriate. Categorical data were summarised as counts and percentages. Continuous variables were compared by independent samples *t* test (normally distributed variables) or Mann–Whitney test (skewed variables). Simple linear regression was applied to investigate correlations between variables. Statistical analysis was performed using GraphPad Prism Version 8 (GraphPad Software).

## Results

### Country and centre characteristics

From a total of 33 countries with national delegates in the AEPC, 31 practitioners with a special interest in paediatric HTx were successfully identified (94%), out of which 28 had been able to complete the survey (90%) at the time of study closure. Demographics of the responding countries are outlined in Table 1, including availability of paediatric HTx services. The median number of paediatric HTx centres per country was 1 (range 0–8). Paediatric HTx centre density in the reporting European countries ranged from 0.0 to 5.35 centres/10 million inhabitants, with a median of 1.16 (Table 1).

**Table 1:** Paediatric heart transplant service and demographics across 28 European countries

**Country**	**Population** *(millions)*	**HTx centres** *(n)*	**HTx centre density** *(per 10 million inhabitants)*	**Nominal GDP** *(billion USD)*	**Nominal GDP per capita** *(billion USD per million inhabitants)*
Austria	9.05	2	2.21	479.82	53.02
Belgium	11.63	2	1.72	609.89	52.44
Bosnia/Herzegovina	3.26	0*	0.00	23.36	7.17
Bulgaria	6.91	1*	1.45	89.53	12.96
Croatia	4.04	1	2.48	69.46	17.19
Czechia	10.72	1	0.93	296.24	27.63
Denmark	5.81	1	1.72	399.10	68.69
Estonia	1.32	0*	0.00	37.20	28.18
Finland	5.55	1	1.80	297.62	53.62
France	65.4	6	0.92	2936.70	44.90
Germany	83.9	8	0.95	4256.54	50.73
Hungary	9.64	1	1.04	177.30	18.39
Iceland	0.34	0*	0.00	27.87	81.96
Ireland	5.00	0*	0.00	516.15	103.23
Italy	60.38	6	0.99	2058.33	34.09
Latvia	1.87	1	5.35	40.27	21.53
Netherlands	17.16	1	0.58	1013.60	59.07
Norway	5.45	1	1.83	541.94	99.44
Poland	37.80	2	0.53	699.56	18.51
Portugal	10.17	2	1.97	251.92	24.77
Romania	19.13	1	0.52	312.49	16.34
Slovenia	2.08	1	4.81	63.65	30.60
Slovakia	5.46	1	1.83	118.43	21.69
Spain	46.77	6	1.28	1435.56	30.69
Sweden	10.15	2	1.97	621.24	61.21
Switzerland	8.70	3	3.45	841.97	96.78
Turkey	85.09	7	0.82	692.38	8.14
United Kingdom	67.89	2	0.29	3376.00	49.73

Demographic data for the included 28 European countries. Population and GDP are from the last full year prior to the survey request was sent out (31st of December 2022). Number of paediatric HTx centres are as reported by the respondents of the survey. Population data and data on GDP were readily available and sourced from Statistica.com

*HTx *heart transplantation, *GDP *gross domestic product, *USD* United States dollar

^*^Shared care program for paediatric HTx available

### Transplant services (questions 5–13 + question 45 + questions 47–48)

**Fig. 1:**
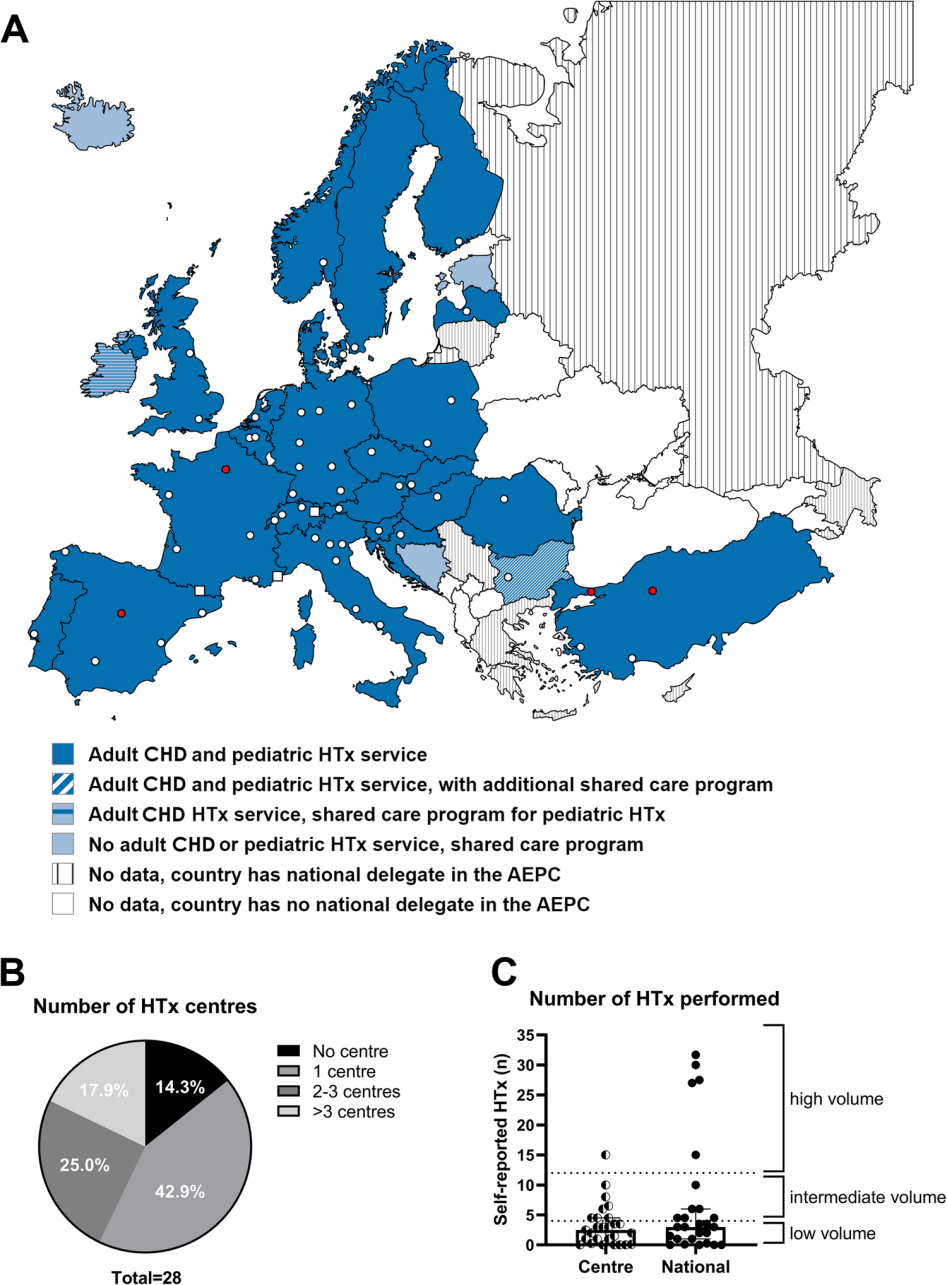
Availability of paediatric and adult congenital HTx services across 28 European countries. Out of 28 surveyed European countries, 24 (86%) had national availability of paediatric and adult congenital HTx services (A). 4 (14%) of the surveyed countries, namely Iceland, Estonia, Ireland, and Bosnia-Hercegovina, had no paediatric HTx services available in their own country, but post-HTx care was available. Ireland had an adult congenital HTx service. These countries were dependent on shared-care programmes with Sweden, Finland, the United Kingdom, and Italy/Turkey, respectively. Interestingly, Bulgaria had a shared-care program with Spain and the United States of America, in addition to a national paediatric HTx program. White dots indicate all cities in Europe with paediatric HTx centres, whereas red dots indicate cities with multiple paediatric HTx centres. White squares indicate three small countries (Andorra, Monaco, and Lichtenstein) from which data was missing. For the number of paediatric HTx centres per country, the most common individual answer was 1 centre/country, although an accumulated 42.9% reported to have > 2 centres (B). Self-reported number of paediatric HTx procedures annually revealed that most centres and countries were classified as having a low volume of transplants (C). Low volume: < 4 transplants/year. Intermediate volume: 4–11.99 transplants/year. High volume: ≥ 12 transplants/year. HTx, heart transplantation; AEPC, Association for European Paediatric and Congenital Cardiology

A map of paediatric and adult congenital transplant service availability is shown in Fig. 1A. Paediatric HTx was a service in twenty-four (86%) of the countries included in this study, while adult congenital transplantation was available in twenty-five (89%). In the four countries with no paediatric HTx centre (14%), a shared-care program was present in all cases. Interestingly, Bulgaria had both adult congenital and paediatric HTx services, with an additional shared-care program. Then, 43% of respondents reported to have one national centre where paediatric HTx was performed (Fig. 1B). Then, 18% reported to have more than three centres where paediatric HTx was performed in their countries. A median of 3 (range 0–31.7) transplants was performed annually in the respective country, on average (Fig. 1C). A median of 2.5 (range 0–15.0) transplants was performed annually at each responding centre, on average (Fig. 1C). Out of 27 respondents reporting their centre volumes, the majority (70%) practised at low-volume centres, 26% practised at intermediate-volume centres, and there was only one (4%) high-volume centre. Classifying each country by volume according to the estimates given by the respondents, most reporting European countries (59.3%) were found to be low-volume countries, and 18.5% were classified as having a high volume of transplants nationally (Fig. 1C). In 22 of 27 centres (81%), there was a dedicated HTx team. In twenty of twenty-seven centres (74%), there was a dedicated transplant advanced nurse practitioner, and in twenty-seven of twenty-seven centres (100%), paediatric cardiologists were reported to be involved in the follow-up of paediatric patients after HTx.

### Transplant listing (questions 14–18 + question 28)

Thirteen (48%) respondents reported that their country followed Eurotransplant protocols and five (19%) followed the Scandiatransplant protocols for listing and organ allocation, of the 27 that answered this question. The remaining nine countries (33%) followed their own national protocols and listing criteria. Seven respondents (25%) reported other restrictions and specific rules for HTx related to age. Six (21%) of those surveyed said that some patients were too young for listing. Specific comments in relation to this theme included “*no specific rules, but we are very selective when listing someone who is younger than 6 months*” and “*only term infants accepted*” for listing. Twenty-three (82%) respondents reported that listed patients could leave the hospital.

### Mechanical circulatory support availability and management (questions 19–27)

**Fig. 2:**
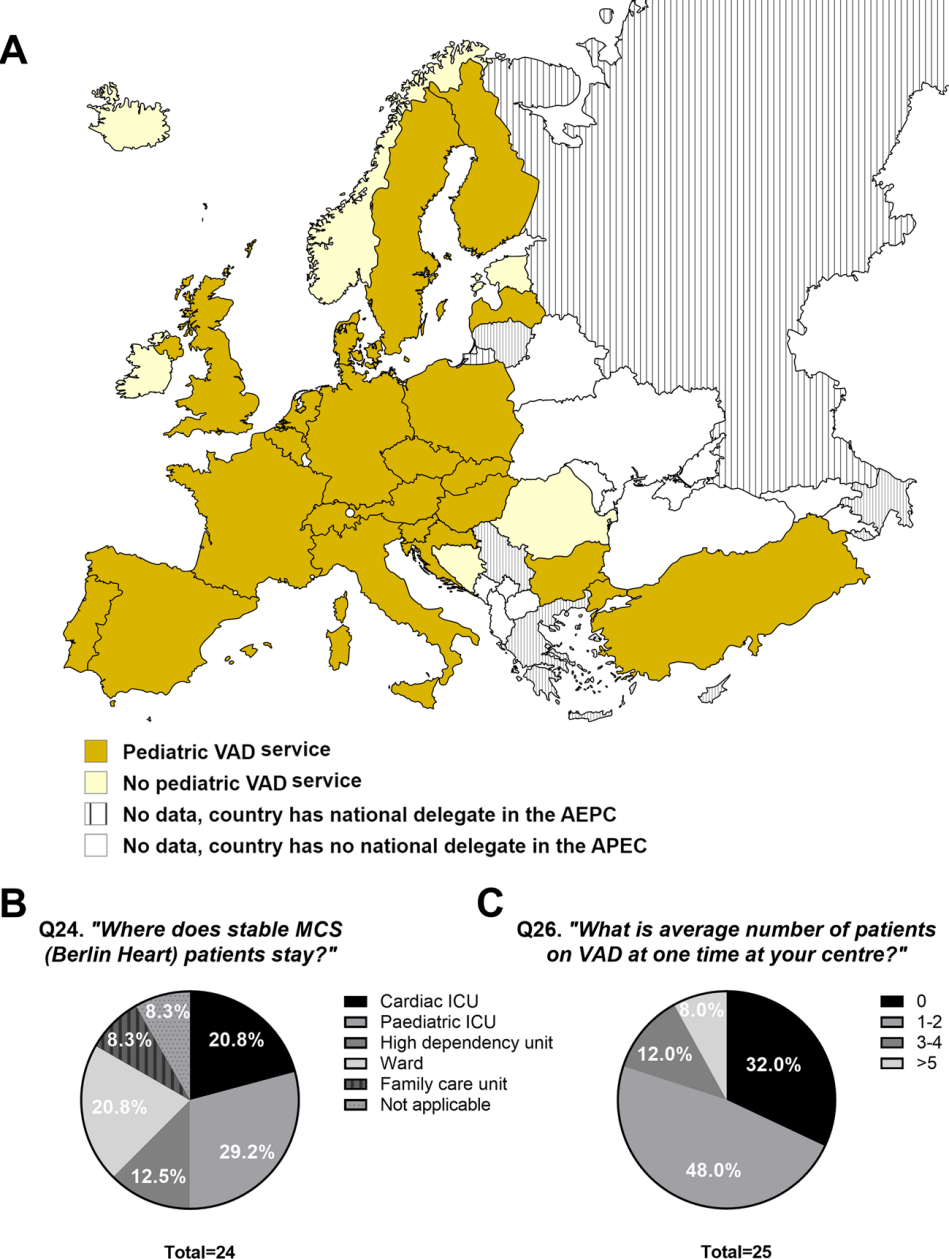
Availability and management of paediatric VAD across 28 European countries. Out of 28 surveyed European countries, 22 (79%) had a national VAD programme (A). Six (21%) of the surveyed countries, namely, Iceland, Estonia, Ireland, Norway, Bosnia-Hercegovina, and Romania, had no paediatric VAD services available in their country. Shared-care programmes for VAD were not investigated or spontaneously mentioned. Two countries (8%, Norway and Romania) lacked a VAD programme although paediatric HTx was available in the country (A). Most commonly, patients on stable MCS (Berlin Heart) were reported to stay at the cardiac or paediatric ICU and only a small number of respondents (8.3%) reported that patients were allowed to stay at family care units (B). When reporting the number of simultaneous VAD patients at any given time, 1–2 patients was the most frequent response, although almost a third (32.0%) reported to have no patients on VAD on average (C). VAD, ventricular assist device; AEPC, Association for European Paediatric and Congenital Cardiology; MCS, mechanical circulatory support; ICU, intensive care unit

The availability of VAD programmes across 28 countries in Europe is outlined in Fig. 2A. Twenty-two countries (79%) had a ventricular assist device (VAD) programme. Two countries (Norway and Romania) had a paediatric HTx programme but no VAD programme (Fig. 2A).

The site of care for stable Berlin Heart patients differed between the 24 countries who reported on this topic (Fig. 2B). In 50% (*n* = 12) of cases, these patients were managed in the cardiac or paediatric intensive care unit (ICU); in three (13%) cases, they were in the high dependency unit; and in five (21%), they were managed on the ward. In two centres (8%), patients were allowed to go to family accommodations for short periods of time. Twelve (46%) of twenty-six respondents reported that there were patients with implantable VADs living at home cared for through their centres.

The average number of patients on VAD (Berlin Heart) support is reported in Fig. 2C. The most common number of patients on VAD at the same time was between 1 and 2 (48%). Participants were then asked what the maximal capacity in their unit for VAD was and invited to provide comments on this. Comments included *“not specified, but volume makes* > *1 theoretical”* and *“there is no official maximal capacity, and it is a case-to-case decision”*.

Of twenty-four participants (86%) who responded to the question whether they would place a VAD in a patient less than five kilograms, 50% (*n* = 12) of those surveyed would place a VAD and 50% would not. Multiple respondents described their centre’s lack of experience with these cases: *“theoretically yes, but it has not been needed for the last 15 years*” and *“lacking expertise”*. Some centres reported a weight less than 5 kg as being “*a relative contraindication*”. Two centres reported that this is used in certain circumstances where there may be a chance of recovered myocardial function *“for a patient younger than 6 months we would be hesitant to place a VAD unless we suspect myocarditis and think there is a chance to recovery”* and *“conditional yes: primarily as bridge to recovery”.*

Twenty-one (81%) of 26 respondents would place a VAD in a patient less than 25 kg while 5 (19%) would not place a VAD in this situation.

### Post-transplant care (questions 28–35)

Twelve respondents (44%) of the 27 who answered the question reported having a standardised national protocol for post-transplant care in their country. In countries with multiple transplant centres (*n* = 12, 43%), 6 respondents (50%) confirmed differences in the management of HTx patients between centres, whereas 3 (25%) were uncertain but believed there were some differences in practice. An example included in the comments *“there are a few differences in induction therapy (ATG vs Basiliximab, no induction), the use of long-term steroids, and maintenance immunosuppression cyclosporin A vs. tacrolimus”.* When there was no standardised national programme available, respondents were asked to describe practice at their own centre.

**Fig. 3:**
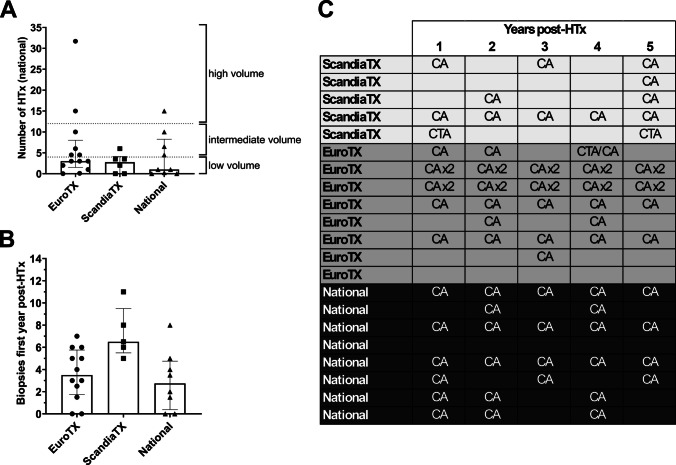
Significant post-HTx management discrepancy between European listing consortia. Based on the reported use of protocols for paediatric HTx listing and organ allocation, the countries were divided into three groups. There was no difference in the estimated number of transplants nationally (A, Kruskal–Wallis test, *p* = 0.5597); however, there was a significant difference in the reported number of biopsies taken during the first year post-transplant (B, Kruskal–Wallis test, *p* = 0.0251), with countries in the Scandiatransplant region taking more biopsies than other countries. Regarding the use of CA and CTA for the screening of CAV, a scheme was created for the use of CA and CTA during the first 5 years post-HTx, in cases where this information was available in the response (C). Of note, two separate centres within Eurotransplant could perform either zero or 10 angiographies during the first 5 years post-HTx. Low volume: < 4 transplants/year. Intermediate volume: 4–11.99 transplants/year. High volume: ≥ 12 transplants/year. EuroTX, Eurotransplant; ScandiaTX, Scandiatransplant; HTx, heart transplantation; CA, coronary angiography; CTA, computed tomography coronary angiogram; CAV, coronary allograft vasculopathy

There were reports of practice variation in the use of biopsies and coronary allograft vasculopathy (CAV) surveillance post-transplant, based on listing consortia (Eurotransplant, Scandiatransplant, or national guidelines). These differences, as well as the number of reported paediatric HTx procedures based on listing consortia (Fig. 3A), are highlighted in Fig. 3. The median number of biopsies performed in the first year in 19 centres reporting their biopsy protocols was 5.5 (range 0–11). A few centres reported different practices in younger/smaller patients “*age related: [age] less than one year – one [biopsy], [age] over one year – 5 [biopsies]*”, “*tend not to biopsy small children, monitor rejection by echo*” and “*average 7, more if rejections; in infants 3*”. Countries within the Scandiatransplant region were found to perform significantly more biopsies compared with other regions, although no common protocol was used in the countries in the Scandiatransplant region (Fig. 3B, p = 0.0123 and *p* = 0.0171).

Twenty-four countries (86%) reported on their practices regarding coronary angiography and intravascular ultrasound (IVUS) for the screening of CAV. Six reporting centres (25%) performed coronary angiography once a year, two centres (8%) performed it twice annually, and two centres (8%) performed no coronary angiography or IVUS. Five centres (21%) specifically reported not using IVUS. Three centres (13%) reported use of CT angiograms as part of their screening protocols for coronary allograft vasculopathy (CAV). Only four of twenty-six centres (15%) performed optical coherence tomography (OCT) at the time of the survey. Figure 3C describes the yearly follow-up protocol for CAV, from the responses that contained such information (*n* = 21), revealing practice heterogeneity.

### Resourcing of paediatric cardiac transplantation programmes (questions 36–44)

#### Financial resources

Only 9 (38%) centres of 24 who responded to this question had dedicated resources for HTx or VAD as part of the hospital budget. Twenty-six respondents answered a question on whether there is a cost for the family having a VAD implanted. Four (15%) reported that health insurance did impact a patient’s access to VAD therapy. Twenty (77%) reported that the VAD was supported by government funding although one respondent made a comment on the unforeseen costs that families are subject to *“…some costs from collateral damage not covered (e.g. sick leave for parents, travel costs, support for siblings)*”.

#### Bed capacity and staffing

Twenty-one of 27 respondents (78%) noted limited intensive care unit (ICU) beds. Fifteen of 27 respondents (56%) also reported shortages of nurses for post-transplant care in the ICU. Fourteen of 26 respondents surveyed (54%) reported that HTx cases impacted negatively on congenital cardiac surgery bed capacity.

### Transplant and mechanical circulatory support centre density does not correlate with population size or country wealth

**Fig. 4:**
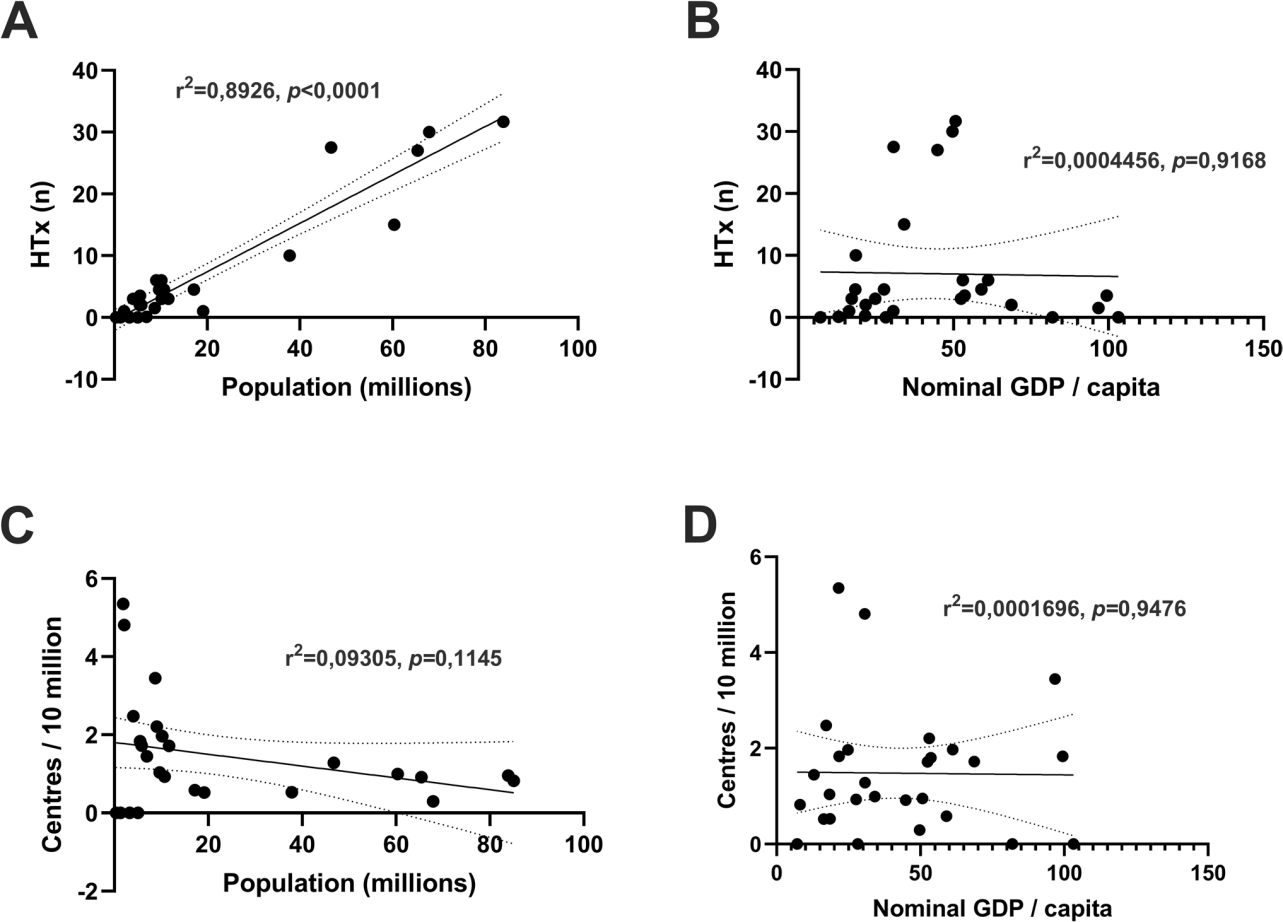
Population size and country wealth does not correlate with paediatric HTx centre density in Europe. Country population and nominal GDP/capita (billion USD per million inhabitants) were plotted against the number of transplant procedures (A, B) and HTx centre density (C, D). Simple linear regressions were fitted to investigate potential correlations. There was a significant correlation between the reported number of transplants and population size (A); however, there was no significant correlation between GDP and the reported number of transplants (B) or centre density and population size or GDP (C, D). A trend towards decreased centre density with increased population was visible; however, it was not significant. There were no trends observed in the correlations with nominal GDP/capita. HTx, heart transplantation; GDP, gross domestic product; VAD, ventricular assist device

The correlations between HTx centre volume and density, population, and GDP are provided in Fig. 4. There was a significant correlation between the reported number of transplants and country size (Fig. 4A, r^2^ = 0.8926, *p* < 0.0001); however, there was no correlation between GDP and the reported number of transplants (Fig. 4B) or centre density and population size or GDP (Fig. 4C and 4D).

### Challenges in the management of pre- and post-transplant patients (question 48)

Twenty-four (86%) of respondents reported on the greatest challenges practitioners faced in the management of pre- and post-transplant patients. The main themes of responses fell under three headings and are reported in Table 2: long waiting list times and lack of donors (*n* = 8, 33%), lack of resources (including beds, staffing, medication, and hospital capacity) reported by 42% (*n* = 10), and low-volume reported by 13% (*n* = 3). Other respondents focused on the complex long-term management of these patients.

**Table 2:** Respondent perception of challenges in the management of pre- and post-transplant paediatric patients

1. Donor pool, recipient status, and waiting list mortality	2. Lack of resources	3. Complex long-term management
“*Unfortunately, waiting time is too long for pre-transplant patients especially small children*”	“*The greatest challenge is the shortage of specialised nurses*.”	“*Main limitations currently psycho-social support*”
“*Referrals from other heart centres mostly at a late stage with beginning multi organ problems, especially those with single ventricle*”	“*Current shortage of cardiac intensive care unit nurses for pre-management mechanical circulatory support…*”	“*Post-transplant follow-ups with committed continuity*”
	**“***No paediatric VAD program in place despite discussions for many years in our institution about medical need.*”	“*Compliance to treatment, mainly in adolescents*”
	“*Underdevelopment of the paediatric heart failure VAD program.*”	

When asked what the main challenges were in the pre- and post-transplant management of paediatric patients, three main themes were identified amongst the responses. Examples from these three main themes are provided above

*VAD* ventricular assist device

### The impact of the COVID-19 pandemic on transplant services (question 49)

The perception of the impact of the COVID pandemic on HTx services differed widely between centres and countries. Twelve (46%) out of the 26 who answered the question reported no or very little impact on paediatric HTx service while other centres reported a significant decrease in the number of transplants and listings. Amongst those who experienced significant alterations in practice during this period, three main themes were identified (please see examples in Table 3): transplant activity, COVID-19 practices, and changes in outpatient management.

**Table 3:** Respondent perception of the impact of COVID-19 on paediatric transplant services

1. Transplant activity	2. COVID-19 practices	3. Outpatient management
“*A disaster*”	“*…not performed if PCR-positive*”	“*Virtual clinics with both shared care programs. Local management of complex rejection AMR. Pivot to perform IVUS angiography and biopsy locally. Annual reviews completed at local centre rather than transplant centre with discussion by virtual clinics*”
“*Certainly has impacted negatively reducing overall activity 25–30%*”	“*In acute COVID infections of recipients we tried to delay HTx at least for two weeks or added targeted therapies. We checked for Covid in the transplanted hearts during surveillance biopsies.*”	“*We used video meetings with some patients when traveling was difficult.*”
“*Numbers of transplantations (children and adults together) went down from* + */ − 25/year to* + */ − 15/year not recovered yet*”		
“*Less donor hearts…*”		

When asked how the COVID-19 pandemic affected paediatric transplant services, three main themes were identified amongst the responses. Examples from these three main themes are provided above

*COVID-19 *coronavirus disease, *PCR *polymerase chain reaction, *HTx *heart transplantation, *AMR *antibody-mediated rejection, *IVUS* intravascular ultrasound

## Discussion

This first survey of availability, practice variation, and resourcing of paediatric HTx care in Europe demonstrated significant variability across the continent. Twenty-four (86%) of the countries surveyed had a paediatric HTx programme. The number of centres ranged from zero to eight per country with a wide range of paediatric HTx centre densities; 0.00–5.35 paediatric HTx centres per ten million inhabitants. Notably, smaller countries often had a higher paediatric HTx centre density, whereas the five countries with the largest populations in Europe (Turkey, UK, Italy, Germany, and France) all displayed a centre density below one per ten million inhabitants. Notably, centre density in the UK was the lowest per ten million inhabitants in Europe (0.29), numbers which exclude the Irish population that also depend on UK paediatric transplant services. A lower centre density does however not entail poor availability, as self-reported centre volumes in these countries far superseded those of smaller European countries.

When countries were classified by centre volume 59.3% were low-volume and only 18.5% were high-volume (≥ 12 transplants annually). This is borne out in data that was previously submitted to the ISHLT suggesting that most European centres were either low- or medium-volume [8]. The thirty-six-year analysis of national paediatric HTx via the United Network for Organ Sharing (UNOS) showed that higher-volume centres were able to transplant higher pre-operative risk patients with similar short-term post-operative outcomes and improved long-term survival [6, 15]. However, recent data from the Scandiatransplant registry has highlighted the excellent outcomes on the waiting list and post-transplant for paediatric HTx in Scandinavia – a region where all centres are considered low-volume, but that share a common waiting list [11, 16, 17]. The authors speculate that free, available healthcare as well as small and highly dedicated multidisciplinary teams are the main factors behind the success of the Scandinavian low-volume transplant programmes and that these factors may be of equal, or even greater, importance than transplant centre volumes.

In the four countries (14%) that did not have a paediatric HTx program, a shared-care transplant programme existed at the time of the survey. These shared-care programmes offer a novel way of overcoming limited resources, whether small-volume centre size or under-funding of transplant or VAD services. It is also interesting to note that changes during the COVID-19 pandemic led to changes in the organisation of some of these shared-care programmes with one respondent remarking that following the pandemic, most follow-up with the transplant centres was conducted with virtual clinics. This shared care arrangement might become more prevalent if European programmes move towards fewer, higher-volume centres.

HTx listing practices vary widely between North America and Europe as demonstrated through successive ISHLT reports [5, 18, 19]. A 2016 study identified that paediatric HTx recipients had the highest waiting list mortality of any solid organ recipients [20]. Our survey highlights the differences in transplant listing practices throughout Europe. There are several different structures for organ listing, procurement and allocation within Europe. Scandiatransplant is an organ listing, exchange programme, and registry with six members (Denmark, Finland, Iceland, Norway, Sweden, and Estonia). In 2023, Scandiatransplant reported both their highest number of donors and their highest number of transplants [21]. Eurotransplant is a network of eight European countries (Austria, Belgium, Croatia, Germany, Hungary, Luxembourg, the Netherlands, and Slovenia) that work in a collaborative framework of organ distribution. In our survey, 19% followed Scandiatransplant and 48% followed Eurotransplant protocols. An example of differing practices/viewpoints was seen in relation to younger patients referred for paediatric HTx. Prior analyses have highlighted the lower infant transplant rate in Europe, as compared to North America [5, 18]. In our survey, six respondents (21%) were of the opinion patients could be too young to be listed. Our data supports the notion that European countries appear to remain more restrictive with infant heart transplant, perhaps due to a small donor pool in this age group, as well as less experience with infant MCS and DCD donors. The European FOEDUS programme allows utilisation of organs that are not suitable for recipients in their default national/international programmes for any reasons [22]. Such organs may be offered to the FOEDUS programme and accepted in another country [22].

The organisation of MCS/VAD programmes was a major theme of this survey. In the current era, only two countries have a paediatric heart transplant programme without a simultaneous VAD programme (e.g. Norway and Romania). The increased use of VAD and the positive one-year survival benefits of VAD support [10, 12] raise questions about the practices in countries that offer a paediatric heart transplant programme without a VAD programme. Fifty-four percent of those surveyed said that cardiac HTx care impacted on congenital heart surgery, and in 50% of centres, VAD patients were cared for in intensive care units. Durable forms of resource allocation and future planning are required to ensure that HTx care does not hinder necessary intensive care and congenital cardiac services and vice versa. Furthermore, the degree of governmental support was high for VAD programmes in most countries. It is however important to acknowledge that these families often spend a significant time on VAD and may not be close to their home and without the certainty of a transplant at the end of that period. Increasing the direct support to the families could therefore be of value.

We identified the greatest practice variation in the post-HTx care of patients. Surprisingly, only 40% of centres had standardised protocols. Interestingly, the UNOS review of thirty-six years of heart transplant outcomes identified standardised practice as being one of the possible benefits to patients of being cared for in a high-volume transplant centre [6]. Many centres used endomyocardial biopsy to screen for rejection with different cut-offs dependent on age and year post-transplant, and most centres screen for CAV with imaging including CA, CTA, IVUS, and OCT. The intervals and number of assessments differed greatly between different countries, in part due to the heterogeneity of the patients. Despite the multiple factors that influence the decision-making around CAV screening, regional exchange might well result in mindful homogenisation of certain post-transplant protocols for the benefit of transplanted children. To exemplify, initiatives to reduce post-transplant coronary angiography/CT angiography and endomyocardial biopsies have been undertaken within the Scandiatransplant region since 2019, as previously reported by the co-authors of this paper [16]. In each transplanted patient, rejection and CAV represent two of the biggest risks to the longevity of their graft and to their long-term health. It seems important to develop universal screening standards that aim to reduce post-HTx morbidity and mortality while also preserving the young person’s quality of life, and comprehensive work has been put in to bring forth such guidelines [23]. The European continent could be vital in providing a collaborative model in developing universal standards for screening post paediatric HTx.

Based on the presented data, we can only speculate on why standardisation of Europe-wide protocols for the listing and management of transplanted children has not yet been achieved; however, a great heterogeneity in languages, political, socioeconomical, and healthcare systems has most likely made any attempts more challenging. On the other hand, clusters of countries have successfully been able to agree on and establish shared-care protocols and collaborative efforts for listing and transplant management, which could serve as model for both countries in Europe and worldwide.

The challenges HTx/heart failure medical professionals identified in our study did not deviate from those expected [19]. Respondents highlighted long waiting times, small donor pools, the complexity of the single-ventricle population, under-resourced services, and the difficulties of post-transplant care.

There are several limitations to this study. First, although there was a high participation rate (90%) in this survey amongst the identified transplant physicians, not all European countries are members of the AEPC. Additionally, not all AEPC member countries participated in the study or could identify their national representative for this subject. Second, participants were largely identified through membership in the cardiac transplantation working group within the AEPC and those not currently members of this group will not have been included in the survey. Third, we relied on the self-reported data from the participants about their centre and country, which naturally are estimates and subject to human error. We asked all participants of the survey to review this paper to ensure data validity, but there may still be inaccuracies included. We acknowledge that these limitations may have introduced systematic bias into our dataset.

## Conclusions

In conclusion, the care of paediatric HTx patients varies widely across Europe. This paper elucidates the differences in transplant service organisation and the care of transplant patients in twenty-eight European countries. These findings could potentially encourage broader open dialogue and facilitate collaboration across European paediatric HTx centres and countries. Increased collaboration could result in the development of standardised listing criteria, improved collective European registry data and creation of standards for screening post-HTx patients, aiming to reduce morbidity and mortality in paediatric end-stage heart failure and HTx patients. Conducting a multinational, retrospective, Europe-wide study on paediatric HTx outcomes would naturally strengthen the speculations of this paper immensely, although this was not feasible with available resources.

## Supplementary Information

Below is the link to the electronic supplementary material.

ESM 1 (DOCX 25.2 KB)

## Data Availability

No datasets were generated or analysed during the current study.

## Abbreviations

HTx: Heart transplantation
ISHLT: International Society for Heart and Lung Transplantation
PHTS: Paediatric Heart Transplant Society
MCS: Mechanical circulatory support
GDP: Gross domestic product
AEPC: Association for European Paediatric and Congenital Cardiology
VAD: Ventricular assist device
UNOS: United Network for Organ Sharing
