# Supplementary material for: Current availability and status of paediatric cardiac transplantation and mechanical circulatory support in twenty-eight European countries
Source: Eur J Pediatr. 2026 Apr 15;185(5):267. doi: 10.1007/s00431-026-06927-1 (PMC13083533; doi:10.1007/s00431-026-06927-1)
Supplement: Supplementary file 1 — (DOCX 25.2 KB) [file 431_2026_6927_MOESM1_ESM.docx]

**Current Availability and Status of Paediatric Cardiac Transplantation and Mechanical Circulatory Support in Twenty-eight European Countries**

**Running Title**

European Paediatric Cardiac Transplantation and Circulatory Support

**Authors**

Oscar van der Have*, Esme Dunne*, Karin Tran-Lundmark, Julie Wacker, Ina Michel-Behnke, Karel Koubsky, Annemarie Krauss, Johan Van Cleemput, Sigurdur Sverrir Stephensen, Michiel Dalinghaus, Dorotea Šijak, Mirko Topalovic, Inguna Lubaua, Zulal Ulger, Vladimiro Vida, Klaus Juul, Christian Balmer, Senka Mesihovic-Dinarevic, José Fragata, Bohdan Maruszewski, Taisto Sarkola, László Ablonczy, Iolanda Muntean, Martin Zahorec, Milind Chaudhari, Thomas Möller, Damien Bonnet, Jacob Simmonds, Zdenka Reinhardt, Colin J. McMahon

**Shared first authors, contributed equally*.

**Corresponding author**: Prof. Colin McMahon MD FRCPI FAHA, CHI Crumlin Dublin Ireland. Email: [cmcmahon992004@yahoo.com](mailto:cmcmahon992004@yahoo.com). Phone: +35314282854.

**Supplemental Information 1. Survey**

1. **What is your name?**

*Free text response*

1. **Which centre do you work in?**

*Free text response*

1. **Which country do you work in?**

*Free text response*

1. **I consent to participate in this survey study.**

*Y/N*

1. **Is adult congenital heart transplantation available in your country?**

*Y/N*

1. **Is paediatric heart transplantation available in your country?**

*Y/N*

1. **If no transplant centre, is there a shared care transplant program (patients sent abroad for transplant)?**

*Y/N*

1. **If a shared care transplant program, which country do you refer patients to?**

*Free text response*

1. **How many paediatric cardiac transplant centres are in your country?**

*Free text response*

1. **Which cities are paediatric cardiac transplant centres located in?**

*Free text response*

1. **How many paediatric heart transplants (average) a year are performed in your country?**

*Free text response*

1. **How many pediatric heart transplants per year (average) are performed at your centre?**

*Free text response*

1. **Who follows paediatric patients after heart transplant?**

*Free text response*

1. **Describe the protocol/listing rules that you follow (Scandiatransplant, Eurotransplant, other)?**

*Free text response*

1. **Are there additional national rules for transplant in your country (between centres etc)?**

*Free text response*

1. **Are there any restrictions/specific rules for cardiac transplantation related to age?**

*Y/N + free text comments*

1. **Are some patients too young for listing?**

*Y/N + free text comments*

1. **Do all listed patients remain in the hospital?**

*Y/N*

1. **Is there a pediatric heart failure VAD program in your country?**

*Y/N*

1. **How many paediatric MCS centres in your country and which cities are they located in?**

*Free text response*

1. **Is Berlin Heart available in your country?**

*Y/N*

1. **For VAD implantation candidates, would you place a VAD in a <5kg patient?**

*Y/N + free text comments*

1. **For VAD implantation candidates, would you place a VAD in a <25kg patient?**

*Y/N + free text comments*

1. **Do stable mechanical support (Berlin Heart) patients go to…**
   1. *Cardiac ICU*
   2. *Paediatric ICU*
   3. *High dependency unit*
   4. *Ward*
   5. *To Ronald McDonald (for short periods)*

*+ comments*

1. **What is the maximal capacity in your unit for VADs (e.g. 2, 5)?**

*Free text response*

1. **What is average number of patients on VAD at one time at your centre?**
   1. *0*
   2. *1-2*
   3. *3-4*
   4. *≥5*
2. **Are there patients on Heartmate at home?**

*Y/N*

1. **If there are several transplant centres in your country, are organs offered in rotation to each hospital or how does this work?**

*Y/N + free text comments*

1. **Is there a standardised national post-transplant protocol for all centres in your country?**

*Y/N + free text comments*

1. **Do centres run their own protocol independent of other centres (in your country)?**

*Y/N + free text comments*

1. **If there is no standardized national post-transplant protocol, please answer the following questions based on the protocol from your own centre?**

*Free text response*

1. **If there are multiple transplant centres in your country is there a difference in management of cardiac transplants between the different centres?**

*Free text response*

1. **How many biopsies in first year?**

*Free text response*

1. **How often do angiography, IVUS?**

*Free text response*

1. **Do you perform OCT (optical coherence tomography) on your patients?**

*Y/N*

1. **Are there limited ICU beds?**

*Y/N*

1. **Are there shortages of nurses for post-transplant care ICU?**

*Y/N*

1. **Are there dedicated resources for cardiac transplant as part of hospital budget?**

*Y/N + free text comments*

1. **Are there dedicated resources for VAD as part of hospital budget?**

*Y/N + free text comments*

1. **Are there dedicated resources for Berlin Heart as part of hospital budget?**

*Y/N + free text comments*

1. **Is there a cost for the family for VAD or is it financed by the government?**

*Free text response*

1. **If there are multiple paediatric VAD centres in your country is there a difference in management of VAD patients between the different centres?**

*Free text response*

1. **Does health insurance impact capacity to have VAD placed?**

*Y/N + free text comments*

1. **Do cardiac transplantation cases impact on congenital cardiac surgical bed capacity?**

*Y/N + free text comments*

1. **For shared care programs how do patients transfer from referee to transplant centre?**

*Free text response*

1. **What are the greatest challenges to your centre in managing pre-, post-transplant patients?**

*Free text response*

1. **Is there a dedicated cardiac transplant team?**

*Y/N*

1. **Is there a dedicated transplant nurse or advanced nurse practitioner?**

*Y/N*

1. **How did the COVID pandemic impact on your transplant service?**

*Free text response*
